# Molecular and functional characterization of the voltage‐gated proton channel in zebrafish neutrophils

**DOI:** 10.14814/phy2.13345

**Published:** 2017-08-03

**Authors:** Adisorn Ratanayotha, Takafumi Kawai, Shin‐ichi Higashijima, Yasushi Okamura

**Affiliations:** ^1^ Laboratory of Integrative Physiology Department of Physiology Graduate School of Medicine Osaka University Suita Osaka Japan; ^2^ Laboratory of Behavioral Neurobiology Department of Biodesign Research Okazaki Institute for Integrative Bioscience Okazaki Aichi Japan

**Keywords:** Hv1, Ion channel, Membrane protein, Phagocyte

## Abstract

Voltage‐gated proton channels (Hv1/VSOP) are expressed in various cells types, including phagocytes, and are involved in diverse physiological processes. Although *hvcn1*, the gene encoding Hv1, has been identified across a wide range of species, most of the knowledge about its physiological function and expression profile is limited to mammals. In this study, we investigated the basic properties of DrHv1, the Hv1 ortholog in zebrafish (*Danio rerio*) which is an excellent animal model owing to the transparency, as well as its functional expression in native cells. Electrophysiological analysis using a heterologous expression system confirmed the properties of a voltage‐gated proton channel are conserved in DrHv1 with differences in threshold and activation kinetics as compared to mouse (*Mus musculus*) Hv1 (mHv1). RT‐PCR analysis revealed that *hvcn1* is expressed in zebrafish neutrophils, as is the case in mammals. Subsequent electrophysiological analysis confirmed the functional expression of DrHv1 in zebrafish neutrophils, which suggests Hv1 function in phagocytes is conserved among vertebrates. We also found that DrHv1 is comparatively resistant to extracellular Zn^2+^, which is a potent inhibitor of mammalian Hv1, and this phenomenon appears to reflect variation in the Zn^2+^‐coordinating residue (histidine) within the extracellular linker region in mammalian Hv1. Notably, the serum Zn^2+^ concentration is much higher in zebrafish than in mouse, raising the possibility that Zn^2+^ sensitivity was acquired in accordance with a change in the serum Zn^2+^ concentration. This study highlights the biological variation and importance of Hv1 in different animal species.

## Introduction

Encoded by *hvcn1*, the voltage‐gated proton channel (Hv1/VSOP) is a membrane protein that mediates the rapid movement of protons (H^+^) across the cell membrane (DeCoursey and Hosler 2014; Okamura et al. 2015; Okamura 2017). Interestingly, the molecular structure of Hv1 consists of a voltage sensor domain (VSD), as found in other voltage‐gated ion channels, but without the pore domain. The VSD is thus responsible for both voltage sensing and proton permeation (Lee et al. 2009). Several of the unique characteristics of Hv1 include: (1) high selectivity for protons (Cherny et al. 2003); (2) activity strongly influenced by the pH gradient across the cell membrane (Kapus et al. 1993; Cherny et al. 1995); and (3) suppressibility by zinc ions (Ramsey et al. 2006; Sasaki et al. 2006).

Up to now, the pattern of *hvcn1* expression has been investigated mainly in mammalian cells, including B lymphocytes (Schilling et al. 2002; Capasso et al. 2010), T lymphocytes (Sasaki et al. 2013), basophils (Musset et al. 2008b), human spermatozoa (Lishko et al. 2010), alveolar epithelial cells (DeCoursey and Cherny 1995), and osteoclasts (Mori et al. 2003). Hv1 function has been particularly well characterized in neutrophils. When these cells generate reactive oxygen species (ROS) during respiratory bursts, electrons are transported from the cytoplasm, leaving an excess of protons inside the cells (DeCoursey 2003a; El Chemaly et al. 2010). Hv1 exports these excessive protons to maintain the appropriate intracellular microenvironment and support ROS generation. Notably, however, the pattern of Hv1 expression differs among distantly related species. For example, Hv1 currents were first described in 1982 in snail giant neurons (Thomas and Meech 1982), but Hv1 has never been detected in mammalian neurons (Okochi et al. 2009; Wu et al. 2012). Likewise, there is no definitive evidence as to whether Hv1 is expressed in phagocytes in species other than mammals. In this study of Hv1 function in vertebrates, we focused on the physiological function and gene expression of Hv1 in zebrafish (*Danio rerio*; DrHv1). Zebrafish is an excellent animal model, owing in part to their transparency at early developmental stages (Kimmel et al. 1995). This enables noninvasive in vivo monitoring and analysis of the physiological function of cells. We demonstrated that the molecular architecture of DrHv1 is similar to other known Hv1 proteins and preserves their key biophysical properties. In addition, by combining molecular biological and electrophysiological techniques, we showed that Hv1 is also expressed in zebrafish neutrophils.

A related area of focus for us was the sensitivity of DrHv1 to extracellular Zn^2+^, which is abundant throughout the body and is essential for a number of cell functions (Roohani et al. 2013; Zhao et al. 2016). For example, Zn^2+^ in cortical neurons is reportedly important for synaptic plasticity and memory formation (Pan et al. 2011). In mammals, Zn^2+^ potently inhibits Hv1 activity, and the Zn^2+^ sensitivity of Hv1 may be related to the proper function of human spermatozoa and mammalian macrophages. Hv1 expressed in spermatozoa is suppressed by enriched Zn^2+^ in the seminal fluid to maintain cellular quiescence prior to ejaculation (Lishko et al. 2010). In macrophages, Hv1 function is regulated by Zn^2+^ levels inside phagosomes in association with ROS production and pathogen elimination (Subramanian Vignesh et al. 2013). In nonmammalian species, however, Zn^2+^ sensitivity of Hv1 and its importance to proper cell function has not been comprehensively investigated. We, therefore, compared the Zn^2+^ sensitivity of DrHv1 with that of mouse (*Mus musculus)* Hv1 (mHv1) to gain fundamental understanding of the physiological function of nonmammalian Hv1 orthologs. DrHv1 showed lower sensitivity to Zn^2+^ than mHv1 and, most remarkably, this difference in Zn^2+^ sensitivity may have a direct correlation with the difference in serum Zn^2+^ concentrations between the two species. This study expands our knowledge of the biological diversity of Hv1 among animal species.

## Material and Methods

### Zebrafish maintenance

RIKEN wild‐type (RW) zebrafish were obtained from RIKEN Brain Science Institute (Saitama, Japan) via an application from the CoMIT Common Fundamental Technology Department, Osaka University (Osaka, Japan). *Tg(mpx:EGFP)* ^*uwm1*^ transgenic zebrafish, which express EGFP in phagocytic lineage cells (Mathias et al. 2006), were purchased from the Zebrafish International Resource Center (ZIRC) (Oregon, USA). The fish were fed brine shrimp twice daily and acclimated in the aquatic facility with a continuous circulation system constantly maintained at 28°C under automatic control with a 14 h/10 h light/dark cycle. All experiments were conducted in agreement with the guidelines of the Animal Care Facility of Osaka University.

### cDNA of DrHv1

cDNA encoding DrHv1 from zebrafish testis was cloned using RT‐PCR. The nucleic acid sequence is identical to the DNA reference **NM_001002345** in the NCBI Databank. For mammalian cell expression, the coding region was subcloned into pIRES‐EGFP plasmid between the XbaI and BamHI restriction sites.

### Cell culture and transfection

HEK293T cells were cultured in DMEM high glucose (Wako, Japan) containing 10% FBS at 37°C under 5% CO_2_. Transient expression was achieved by transfecting DrHv1‐containing pIRES2‐EGFP plasmid into cells using Polyfect^®^ Transfection Reagent (Qiagen, Germany) according to the manufacturer's instructions.

### Cell dissociation of zebrafish larvae

*Tg(mpx:EGFP)* ^*uwm1*^ transgenic zebrafish (Mathias et al. 2006) were used for cell dissociation. Approximately 200 larvae were collected at 3 days after fertilization and dissociated mechanically in Leibovitz's L‐15 medium without phenol red (Invitrogen, USA), as described in the Zebrafish Book Protocol (Westerfield 2000). The dissociated cells were transferred to a culture dish and incubated at 28°C for at least 10 min before starting the experiment. BD FACSAria™ II (BD Bioscience, USA) was used to isolate EGFP‐positive cells from the dissociated cell suspension.

### Electrophysiology

Whole‐cell patch clamp recordings were made using DrHv1‐expressing HEK293T cells. An AxoPatch™ 200B amplifier (Molecular Devices, USA) and pCLAMP™ 10.3 (Molecular Devices, USA) were used for electrical stimulation and data acquisition. Glass patch pipettes were made from 100 *μ*L Calibrated Pipettes (Drummond Scientific Company, USA). The bath solution contained 75 mmol/L N‐methyl‐D‐glucamine (NMDG), 1 mmol/L CaCl_2_, 1 mmol/L MgCl_2_, and 180 mmol/L HEPES (pH 7.0–8.0) or 180 mmol/L MES (pH 6.0–6.5). The pipette solution contained 65 mmol/L NMDG, 3 mmol/L MgCl_2_, 1 mmol/L EGTA, and 183 mmol/L HEPES (pH 7.0) or 110 mmol/L MES (pH 6.0). The designated pH was titrated using methanesulfonic acid for acidification or NMDG for alkalization. The osmolality of all solutions was adjusted to approximately 0.300 Osm/kgH_2_O using glucose. The holding potential was set at −80 mV unless otherwise noted in the figure legend. A series of 3‐s depolarizing step pulses were applied from the holding potential to 100 mV in 10‐mV increments. A tail current protocol was used to measure the reversal potential: depolarizing voltage pulses to 60 mV were applied for 200 msec, after which the membrane was repolarized to a level ranging from −80 to 100 mV for 200 msec. A DAD Superfusion System (ALA Scientific Instruments, USA) was applied for rapid perfusion of bath solution during measurements of the reversal potential, pH‐dependent gating, and Zn^2+^ sensitivity. All experiments were done at room temperature (23–26°C).

*Tg(mpx:EGFP)* ^*uwm1*^ transgenic zebrafish were used for recording proton currents in neutrophils. The instruments and patch clamp device settings were identical to those used with HEK293T cells, except the holding potential was set at −60 mV, and a series of 1‐s depolarizing step pulses were applied from −60 mV to 100 mV in 20‐mV increments. This protocol was also used in HEK293T cells for comparative experiments with neutrophils.

### Analysis of *hvcn1* expression in zebrafish

Adult RW zebrafish were anesthetized for 5 min using 0.2 mg/L ethyl 3‐aminobenzoate methanesulfonate (Tricaine) solution (Sigma‐Aldrich, USA) and then perfused transcardially with RNase‐free PBS to avoid contamination by blood cells. Ovaries (in female), testis (in male), spleen, liver, intestine, kidney, heart, gill, eyeballs, and brain were carefully dissected. Total RNA was purified from each tissue using TRIzol^®^ LS reagent (Invitrogen, USA) according to the manufacturer's protocol with volume adjustment for the small size of zebrafish tissue samples. Total RNA (200 ng) extracted from each organ was reverse transcribed using SuperScript^®^ III First‐Strand Synthesis System (Invitrogen, USA). For semiquantitative RT‐PCR, fragments of DrHv1 and *β*‐actin were amplified from cDNA using the primer sets listed in Table 1.

**Table 1:** Primers used in this study

Target gene	Oligonucleotide sequences (5′ – 3′)	
*hvcn1*	Sense Antisense	CCTTCTCACATTCTTCATGG TTCTGCTCTTTAAGCTCATTG
*β‐actin*	Sense Antisense	CGAGCTGTCTTCCCATCCA TCACCAACGTAGCTGTCTTTCTG
*aanat1*	Sense Antisense	GGACCAGGACCGTCTGACT CTGCAAGTAACGCCACAAGA
*mef2a*	Sense Antisense	GGCTCTCCAGGGCTCTCTAT CATTCTGGCTGGTGTTGATG
*mitfa*	Sense Antisense	GCAGCAGAAAGCAAAAGAGC GGCTGGAAGAAGCTACAACG
*mpx*	Sense Antisense	GCTGCTGTTGTGCTCTTTCA TTGAGTGAGCAGGTTTGTGG

To assess DrHv1 expression in zebrafish neutrophils, EGFP‐positive cells freshly isolated from *Tg(mpx:EGFP)* ^*uwm1*^ transgenic zebrafish larvae 3 days after fertilization were subjected to FACS analysis. Total RNA was extracted and cDNA synthesized, after which RT‐PCR was performed using the same primer sets used for the tissue analysis. Additional primer sets for positive and negative controls are also listed in Table 1.

### Blood sampling and analysis of serum Zn^2+^ concentration

Blood samples were collected from adult RW zebrafish (*n* = 10) using the spinning method described by Babaei et al. (2013). Blood samples were collected from mice via the tail vein (*n* = 6). Blood samples from the African clawed frog (*Xenopus laevis*) were collected directly via cardiocentesis (*n* = 2). All samples were immediately placed on ice and centrifuged at 4°C for 15 min at 13,700 *g*, 2000 *g*, and 18,200 *g* for zebrafish, mouse, and African clawed frog, respectively. Zn^2+^ concentrations were measured in serum samples using a Metalloassay Zn LS kit (Metallogenics Co., Japan) following the manufacturer's instruction.

### Data analysis and statistics

Electrophysiological data were analyzed using Clampfit Software 10.7 (Molecular Devices, USA), and the statistical results are presented as means ± SD. Graphs and fitting data were calculated using Igor Pro Software 6.37 (Wavemetrics, USA). Statistical analysis was performed using Prism 6 (Graphpad Software, USA).

## Results

### Electrophysiological characteristics of DrHv1

**Figure 1:**
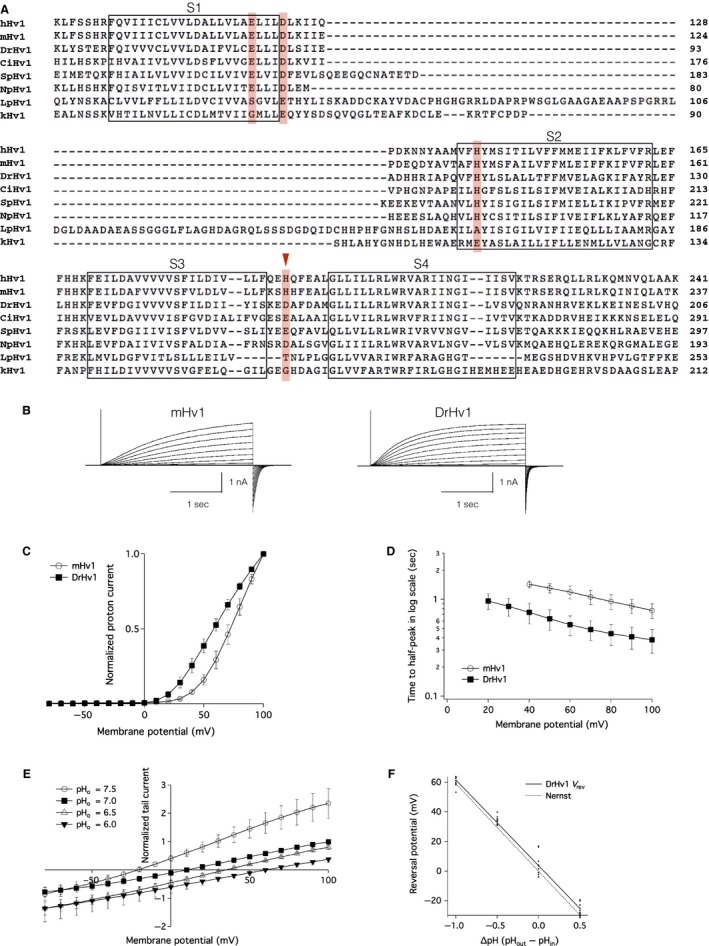
Characterization of zebrafish Hv1. (A) Multiple amino acid alignment of Hv1 from human (*Homo sapiens*, hHv1), mouse (*Mus Musculus*, mHv1), zebrafish (*Danio rerio*, DrHv1), sea squirt (*Ciona intestinalis*, CiHv1), sea urchin (*Strongylocentrotus purpuratus*, SpHv1), insect (*Nicoletia phylophila*, NpHv1), and two species of dinoflagellates (*Lingulodinium polyedrum*, LpHv1; *Karlodinium veneficum*, kHv1;). Putative transmembrane helices (S1–S4) are indicated by the boxes. Zn^2+^‐coordinating residues are shown in red. This figure contains partial sequences focused on the main components of the Hv1 channel. Full‐length sequences can be found in Fig. S1. (B) Representative proton current traces recorded from mHv1‐ and DrHv1‐expressing HEK293T cells (left and right, respectively). The holding potential was ‐80 mV. A series of 3‐s depolarizing voltage pulses were stepped from −80 mV to 100 mV in 10‐mV increments. Both the extracellular and intracellular pHs (pH_o_ and pH_i_, respectively) were 7.0. The vertical scale bar indicates the current amplitude and the horizontal scale bar indicates the duration. (C) Normalized current–voltage relationships of mHv1 (empty circles; *n* = 5) and DrHv1 (filled squares; *n* = 5). Data are presented as means ± SD. (D) T‐half values for the activation phase of mHv1 (empty circles; *n* = 5) and DrHv1 (filled squares; *n* = 5) plotted against the membrane potentials. Note that the *y*‐axis is calibrated in log scale. (E) Normalized current–voltage relationship recorded using a tail current protocol at pH_o_ 7.5 (empty circles), 7.0 (filled squares), 6.5 (empty triangles) and 6.0 (filled triangles) and pH_i_ fixed at 7.0 (*n* = 10). Tail currents were measured during the second depolarizing step from −80 mV to 100 mV. (F) Relationship between reversal potential (V_rev_) and pH gradient across the cell membrane (ΔpH) (*n* = 10). Solid line is the fitting result from the averaged V_rev_. The dotted line shows the predicted values from the Nernst equilibrium equation for proton conductance.

Multiple amino acid sequence alignment of DrHv1 with Hv1 from other species (Figs. 1A and S1) showed a common molecular architecture with the following features conserved among them: (1) the four putative transmembrane helices (S1–S4) of the VSD (Ramsey et al. 2006; Sasaki et al. 2006); (2) three positively charged Arg (R) residues in the S4 helix, which are essential for the voltage sensing mechanism (Gonzalez et al. 2013); and (3) a coiled‐coil structure at the C‐terminus (Lee et al. 2008). The N‐terminus of DrHv1 is shorter than those of the mammalian orthologs, but it is comparable to other teleosts (Fig. S2), and contains a short amphipathic helix homologous to the S0 region adjacently preceding the S1 helix (Takeshita et al. 2014).

We examined the basic electrophysiological properties of DrHv1 in a whole‐cell patch clamp analysis of DrHv1‐expressing HEK293T cells. As expected, DrHv1 showed outward proton currents in response to depolarizing voltage steps (Fig. 1B). The normalized current–voltage (I‐V) relationship revealed that DrHv1 is activated at a lower membrane potential than mHv1, as the voltage threshold (V_threshold_) for the activation of DrHv1 is more negative than that for activation of mHv1 (Fig. 1C). To discuss the possibility that the difference in V_threshold_ was due to the incomplete activation of Hv1s, the normalized I‐V relationships were also calculated from the exponentially fitted data of the corresponding actual current traces (Fig. S3). We confirmed that the results from actual and fitted traces were highly equivalent, suggesting that the difference is not due to the different activation kinetics. Furthermore, we noticed that the activation kinetics of DrHv1 are slightly faster than those of mHv1 (Fig. 1B). The T‐half, at which the current reaches its half‐maximum magnitude, was measured to compare the activation rates of DrHv1 and mHv1 (Fig. 1D). The average T‐halves for DrHv1 were 0.74 ± 0.18 sec and 0.38 ± 0.11 sec at membrane potentials of 40 mV and 100 mV, respectively (*n* = 5); for mHv1, the T‐halves were 1.44 ± 0.12 sec and 0.76 ± 0.13 sec at 40 mV and 100 mV, respectively (*n* = 5). Overall, the T‐half for DrHv1 was about half that for mHv1 at all designated membrane potentials.

Reversal potential (V_rev_) measurement at different ranges of extracellular pH (pH_o_) and intracellular pH (pH_i_) (Fig. 1E, F) confirmed that the DrHv1‐derived currents were carried by protons. The measured V_rev_ values for DrHv1 were close to the values predicted by the Nernst equilibrium equation for proton conductance at every specified pH gradient across the cell membrane (ΔpH = pH_o_–pH_i_) (*n* = 10). Thus, DrHv1 functions as a voltage‐gated proton channel.

**Figure 2:**
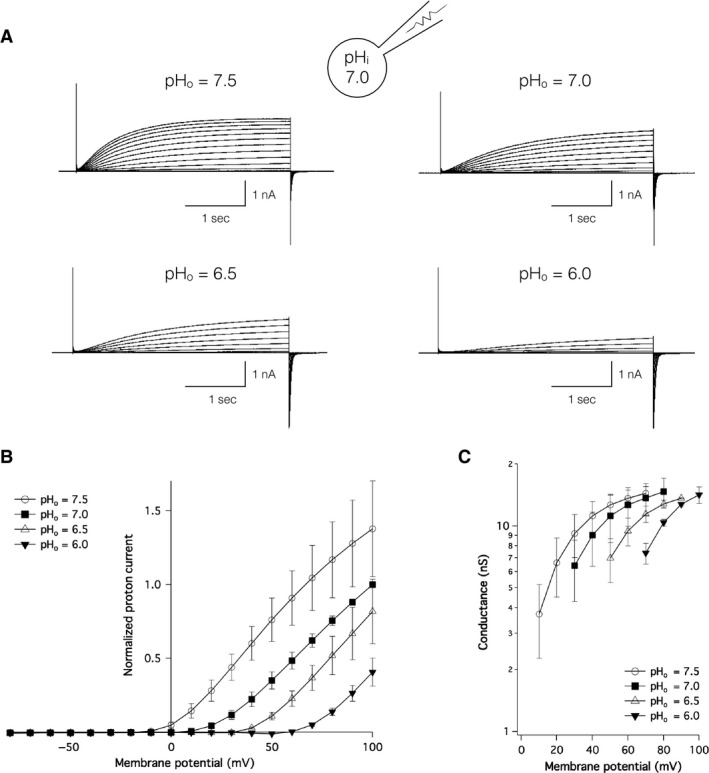
pH‐sensitivity of channel gating of DrHv1. (A) Representative proton current traces recorded from a single DrHv1‐expressing HEK293T cell. The holding potential was −80 mV. A series of 3‐s depolarizing voltage pulses were stepped from −80 mV to 100 mV. pH_i_ was 7.0; pH_o_s were 7.5, 7.0, 6.5, and 6.0. (B) Normalized current–voltage relationships of DrHv1‐derived proton currents recorded at different ΔpHs (*n* = 5). pH_i_ = 7.0; pH_o_s were 7.5 (empty circle), 7.0 (filled squares), 6.5 (empty triangles), and 6.0 (filled triangles). (C) Conductance–voltage relationships of DrHv1 at different ΔpHs (*n* = 5). Data were taken from the same set of cells as in (B).

Because the voltage‐regulated gating of Hv1 in other species is reportedly pH‐dependent (Kapus et al. 1993; Cherny et al. 1995), DrHv1‐derived proton currents were measured within different ΔpHs. The representative traces for DrHv1‐derived proton currents (Fig. 2A) recorded at pH_o_ ranging from 6.0 to 7.5 and with pH_i_ fixed at 7.0. The traces demonstrated that the general appearance of current traces changed upon alteration of the ΔpH. The normalized I‐V relationships at several designated ΔpHs (Fig. 2B) clearly validated the pH‐dependency of the DrHv1 activities: a positive ΔpH resulted in greater current amplitudes, whereas a negative ΔpH resulted in smaller ones. In addition, when the conductance measured at different ΔpHs was plotted against the membrane potential, it also showed a clear pH‐dependent shift (Fig. 2C), which confirmed that pH dependence of the voltage‐regulated gating is a key feature of DrHv1.

### Profile of *hvcn1* expression in zebrafish

**Figure 3:**
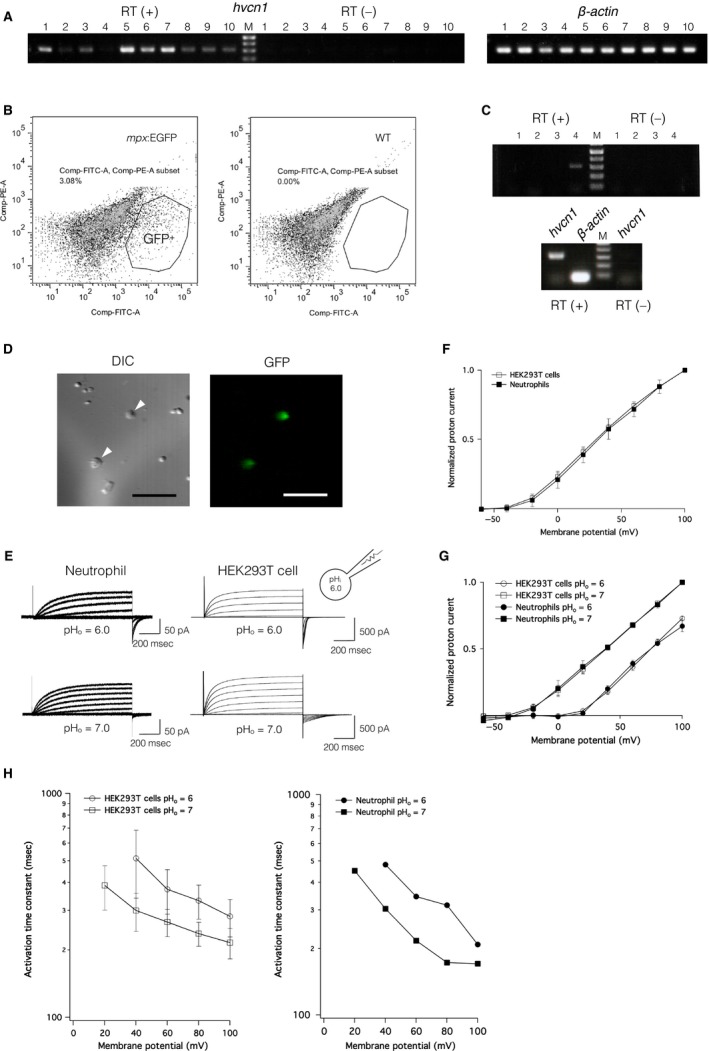
*hvcn1* expression profile and native voltage‐gated proton channel currents recorded from neutrophils. (A) RT (+), *hvcn1* expression in adult zebrafish tissues: (1) ovary, (2) spleen, (3) liver, (4) intestine, (5) kidney, (6) heart, (7) gill, (8) eyeballs, (9) brain, and (10) testis. RT (‐), negative control for all organs in the same order. *β‐actin* served as the positive control. (B) Flow cytometric analysis of cell populations from *Tg(mpx:EGFP)* ^*uwm1*^ transgenic zebrafish (left) and wild‐type zebrafish (right). EGFP‐positive cells are indicated within the surrounded area. (C) *Upper*, RT‐PCR results for zebrafish neutrophils isolated using FACS. (1) *aanat1*, expressed only in retina, (2) *mef2a*, expressed in heart, muscle and somites during zebrafish embryogenesis, (3) *mitfa*, expressed in pigmented cells and retinal pigmented epithelium, (4) *mpx*, expressed in phagocytes. (1)– (3) show negative results, whereas (4) shows positivity for the PCR product. *Lower*, RT‐PCR result for *hvcn1* in zebrafish neutrophils. RT (‐), negative control; *β‐actin*, positive control. (D) EGFP‐positive neutrophils isolated from *Tg(mpx:EGFP)* ^*uwm1*^ transgenic zebrafish. Arrowheads indicate zebrafish neutrophils (left) corresponding to EGFP‐positive cells (right). Scale bar = 50 *μ*m. (E) Representative DrHv1‐derived proton current traces recorded from a single zebrafish neutrophil (left) and a single DrHv1‐expressing HEK293T cell (right). A 1‐s depolarizing voltage protocol was stepped from −60 mV to 100 mV in 20‐mV increments. pH_i_ was 6.0; pH_o_s were 6.0 and 7.0. (F) Normalized current–voltage relationships of DrHv1‐derived proton currents recorded from DrHv1‐expressing HEK293T cells (empty squares; *n* = 8) and zebrafish neutrophils (filled squares; *n* = 8). pH_i_ was 6.0, and pH_o_ was 7.0. (G) Normalized current–voltage relationships of DrHv1‐derived proton currents recorded from DrHv1‐expressing HEK293T cells (empty symbols; *n* = 4) and zebrafish neutrophils (filled symbols; *n* = 2) at different ΔpHs. pH_i_ was 6.0; pH_o_s were 6.0 (circles) and 7.0 (squares). (H) The activation time constant of DrHv1‐derived proton current measured in DrHv1‐expressing HEK293T cells (left) and a representative zebrafish neutrophil (right). pH_i_ was 6.0; pH_o_s were 6.0 (circles) and 7.0 (squares). Note that in both panels the *y*‐axis is calibrated in log scale. The holding potential was −60 mV throughout the data in Figure 3.

RT‐PCR analysis of zebrafish tissue samples showed significant expression of *hvcn1* in kidney, gill, and heart (Fig. 3A). It also showed small, but reproducible, signals in ovaries, liver, eyeballs, brain, and testis. In contrast, negative controls, which did not contain reverse transcriptase, did not show a corresponding PCR product. This result indicates *hvcn1* is expressed in a wide range of tissues in zebrafish. We therefore speculated that *hvcn1* is also expressed in zebrafish immune cells, considering the expression profile and significant role of Hv1 orthologs in mammals (Okochi et al. 2009; El Chemaly et al. 2010). To test that idea, we used *Tg(mpx:EGFP)* ^*uwm1*^ transgenic zebrafish (Mathias et al. 2006), which express EGFP protein in phagocytic lineage cells, primarily in neutrophils (Fig. 3D). Flow cytometry of dissociated cells from zebrafish larvae collected at 3 days after fertilization shows a distinct population of EGFP‐positive cells in transgenic zebrafish not seen in RIKEN wild‐type (RW) fish (Fig. 3B). As expected, RT‐PCR analysis of the isolated EGFP‐positive cells clearly showed expression of *mpx* (Fig. 3C), but the cells were negative for *aanat1* (retina), *mef2a* (heart, muscle, and somite during zebrafish embryogenesis) and *mitfa* (pigmented cell and retinal pigmented epithelium). Significant expression of *hvcn1* was also observed in the isolated EGFP‐positive cells (Fig. 3C), indicating that zebrafish neutrophils express *hvcn1*.

Whole‐cell patch clamp analysis of DrHv1 activity in zebrafish neutrophils revealed outward proton currents that were elicited in response to depolarizing voltage steps and were affected by the ΔpH in a manner similar to the currents observed in DrHv1‐expressing HEK293T cells (Fig. 3E). Comparison of the normalized I‐V relationships demonstrated a high equivalence between DrHv1‐derived proton currents recorded from zebrafish neutrophils and DrHv1‐expressing HEK293T cells at both pH_o_ 6.0 and pH_o_ 7.0 (Fig. 3F, G). We also determined the activation time constant (*τ*) of DrHv1 in both native cells and the heterologous HEK293T cell expression system (Fig. 3H). Again, the activation time constant in neutrophils was shifted with changes in extracellular pH, as was the case with DrHv1‐expressing HEK293T cells. These electrophysiological findings appear to confirm expression of DrHv1 in zebrafish neutrophils.

### DrHv1 is more resistant to extracellular Zn^2+^ than mHv1

**Figure 4:**
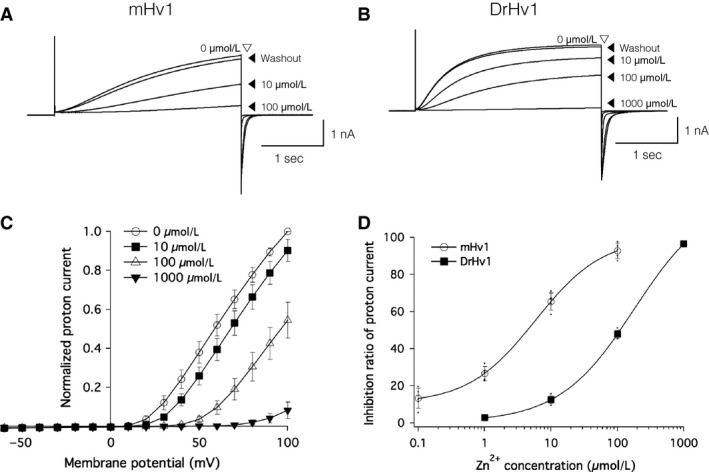
Zn^2+^ sensitivity of DrHv1 is lower than that of mHv1. (A and B) Zn^2+^ sensitivities of mHv1 (A) and DrHv1 (B). Representative current traces recorded from a single mHv1‐ or DrHv1‐expressing HEK293T cell with the indicated concentrations of Zn^2+^ in the bath solution. Currents were measured at a membrane potential of 100 mV for 3 sec. The Zn^2+^ concentration was changed in the sequence (in *μ*mol/L) 0, 10, 100, and 0 (washout) for mHv1 or 0, 10, 100, 1000, and 0 (washout) for DrHv1. Both pH_i_ and pH_o_ were 7.0. (C) Normalized current–voltage relationships of DrHv1‐derived proton currents with the indicated Zn^2+^ concentrations in the bath solution. The holding potential was −60 mV. A 3‐s depolarizing voltage pulse was stepped from −60 mV to 100 mV. Both pH_o_ and pH_i_ were 7.0. (D) Percent inhibition of mHv1‐ and DrHv1‐derived proton currents (*n* = 5, 5, respectively) at different concentrations of Zn^2+^ in the bath solution. Data are shown as means ± SD and the curve was fitted with the Hill Equation (Igor Pro 6.37, Wavemetrics, USA)

**Figure 5:**
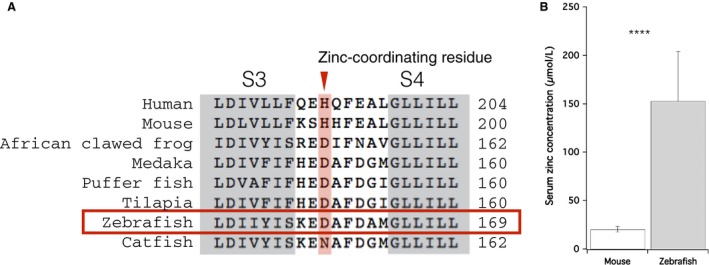
Serum Zn^2+^ concentration in zebrafish. (A) Partial amino acid sequence alignments at the S3‐S4 linker region of Hv1 from human, mouse, African clawed frog, and selected teleosts. Zn^2+^‐coordinating residues (histidine, H) are shown in a box (red). (B) Comparison of the total serum Zn^2+^ concentrations in mouse (left) and zebrafish (right). Data were shown as means ± SD. ****P < 0.0001, unpaired t‐test.

Because extracellular Zn^2+^ is a well‐known inhibitor of Hv1 in other species (Cherny and DeCoursey 1999; Decoursey 2003b), we also examined the effect of Zn^2+^ on DrHv1 currents. We found that extracellular Zn^2+^ suppressed DrHv1‐derived currents in a concentration‐dependent manner (Fig. 4B–D) with an IC_50_ of approximately 100 *μ*mol/L. When compared with mHv1 (Fig. 4A), DrHv1 showed greater resistance to extracellular Zn^2+^, shifting the concentration‐response curve by about one order of magnitude (Fig. 4D). Previous studies demonstrated four Zn^2+^‐coordinating residues in mHv1 – that is, Glu 115 (E115), Asp 119 (D119), His 136 (H136), and H189 – which are critical for the regulation of Zn^2+^ inhibition (Ramsey et al. 2006; Takeshita et al. 2014). Multiple sequence alignments showed that E115, D119, and H136 of mHv1 are conserved in almost all selected species (Figs. 1A, S1 and S2). In contrast, although a H189‐homologous residue is found in human Hv1 (hHv1) and mHv1, but not in Hv1 channels from teleosts, including zebrafish (Fig. 5A and S2). We suggest this replacement of His189 in mHv1 with Asp in DrHv1 likely accounts for the difference in Zn^2+^ sensitivity between them.

### Serum Zn^2+^ concentrations are much higher in zebrafish than mice

Although DrHv1 and mHv1 have different sensitivities to extracellular Zn^2+^, both are expressed in phagocytes, which are one of the cellular components circulating in the blood. Assuming the serum Zn^2+^ concentration has an impact on the physiological function of Hv1, it would be important to compare the serum Zn^2+^ concentrations in mice and zebrafish. After collecting blood from adult animals of both species and measuring the total serum Zn^2+^ concentration, we found that it is 20.6 ± 2.77 *μ*mol/L (*n* = 6) in mouse, which is consistent with earlier studies (Boobis and Hartley 1981). In contrast, the serum Zn^2+^ concentration in zebrafish is 153 ± 51.2 *μ*mol/L (*n* = 10). We suggest the eight times higher Zn^2+^ concentration (Fig. 5B) likely reflects the difference in Zn^2+^ sensitivity between DrHv1 and mHv1. We also measured the serum Zn^2+^ concentration in the African clawed flog (*Xenopus laevis*). Its Hv1 ortholog also does not contain a His within the S3‐S4 linker region, and at 191.8 ± 22.1 *μ*mol/L (*n* = 2), its serum Zn^2+^ concentration is comparable to that in zebrafish (Table 2).

**Table 2:** Zn^2+^ concentration in blood serum (*μ*mol/L)

Species	Serum [Zn^2+^] (*μ*mol/L)	Histidine	*n*	Reference
Human (hHv1)	12.9–24.3 16.7–19.9	+		(Yanagisawa 2004) (Flomenbaum et al. 2006)
Mouse (mHv1)	20.6 ± 2.8	+	6	This study
African clawed frog (XlHv1)	191.8 ± 22.1	‐	2	This study
Zebrafish (DrHv1)	153 ± 51.2	‐	10	This study
Channel catfish (IpHv1)	405.5 ± 9.8	‐	4	(Cort et al. 1995)

## Discussion

In this study, we used electrophysiological and molecular biological techniques to investigate the function and expression profile of DrHv1, an Hv1 ortholog in zebrafish. Our results confirmed the shared features of the proton channel function and gene expression pattern of DrHv1 and mammalian Hv1 orthologs. However, we also found differences between the electrophysiological properties and Zn^2+^ sensitivity of DrHv1 and mammalian Hv1, which may be related to the biological function of native Hv1 in teleost species.

### Electrophysiological properties of DrHv1 as a voltage‐gated proton channel

The amino acid sequence of DrHv1 shared a high degree of molecular identity with mammalian Hv1 channels, including a VSD with four transmembrane regions and a coiled‐coil structure at the C‐terminus (Fig. 1A). As anticipated, whole‐cell patch clamp analysis in a heterologous expression system confirmed DrHv1's voltage‐gated proton channel activity (Fig. 1B, C). DrHv1 also exhibited the following biophysical properties of Hv1 characterized in other species: (1) proton‐selectivity, as indicated by V_rev_ values close to those predicted from the Nernst equilibrium equation for proton (Fig. 1F) (Cherny et al. 2003); (2) pH‐dependent gating, shown by the current and conductance shift following alteration of the ΔpH (Fig. 2B, C) (Kapus et al. 1993; Cherny et al. 1995); and (3) Zn^2+^ sensitivity, as indicated by concentration‐dependent suppression of channel activity by extracellular Zn^2+^ (Fig. 4C) (Ramsey et al. 2006; Sasaki et al. 2006).

While preserving the common features of Hv1, DrHv1 also shows the following electrophysiological properties that are distinct from those of mHv1: (1) a more negative V_threshold_ (Fig. 1C) and (2) activation kinetics that are somewhat faster at all tested membrane potentials (Fig. 1D). It is noteworthy that the activation kinetics of Hv1 markedly vary among the different species that have been investigated thus far (Smith et al. 2011; Taylor et al. 2011, 2012; DeCoursey 2013; Chaves et al. 2016; Sakata et al. 2016). For example, the activation kinetics of SpHv1, cloned from sea urchin (*Strongylocentrotus purpuratus*) are approximately 20–60 times faster than mammalian Hv1 kinetics (Sakata et al. 2016). While those studies characterized the electrophysiological properties of Hv1 in invertebrates, this study is the first to characterize the function of Hv1 in nonmammalian vertebrates. Because Hv1 appears to be commonly expressed in neutrophils from vertebrates, as will be discussed in the next section, it is important to focus on their different activation kinetics for comparative physiological understanding of neutrophil function. It is noteworthy that the difference in kinetics between mHv1 and DrHv1 may be related to the difference in their normal body temperatures. Previous studies reported that the Hv1 gating mechanism is highly sensitive to the temperature (DeCoursey and Cherny 1998; Fujiwara et al. 2012); higher temperatures greatly accelerate the activation kinetics. Considering the difference in body temperature between the mouse (homeotherm, 37°C) and zebrafish (poikilotherm, around 28°C), we suggest the activation kinetics of Hv1 in the native phagocytes under normal conditions may not differ much, though this will require future testing.

### DrHv1 is expressed in zebrafish phagocytes

In this study, we used several approaches to examine the pattern of *hvcn1* expression in zebrafish. The expression of *hvcn1* was detected in a wide range of zebrafish tissues (Fig. 3A). Taking into consideration that the kidney is a hematopoietic organ in zebrafish (Davidson and Zon 2004; Murayama et al. 2006), positive RT‐PCR results in kidney and EGFP‐positive cells isolated from *Tg(EGFP:mpx)* ^*uwm1*^ zebrafish larvae (Fig. 3C) support our hypothesis that DrHv1 is expressed in zebrafish immune cells. In mammals, Hv1 reportedly plays a significant role in the immune system (DeCoursey and Cherny 1993; Demaurex et al. 1993; Morgan et al. 2003; Okochi et al. 2009; Petheo et al. 2010). Our electrophysiological analysis also demonstrated for the first time that zebrafish neutrophils are capable of producing voltage‐dependent proton currents (Fig. 3F) that exhibit key features of Hv1 channels (Fig. 3E). In addition, comparison of the normalized I‐V relationships and activation time constants (*τ*) of the proton currents measured in native cells and heterologous expression in HEK293T cells (Fig. 3F–H) indicate the high identity of DrHv1 activity in both systems. Some subtle differences in the activation kinetics of native Hv1 currents were noticed when compared with those in HEK293T cells. These are possibly due to the different protein expression level, or unknown intrinsic cellular properties unique to neutrophils or HEK293T cells (Musset et al. 2008a). Taken together, our results confirm the functional expression of DrHv1 in zebrafish neutrophils and suggest a potential role for Hv1 in teleost phagocytic cells. We therefore conclude that phagocytic expression of Hv1 is conserved from teleosts to mammals.

### Low sensitivity of DrHv1 to extracellular Zn^2+^

It is well‐known that suppression by extracellular Zn^2+^ is a hallmark property of Hv1 currents. In this study, we confirmed the sensitivity of DrHv1 to the extracellular Zn^2+^. Figure 4C shows that a higher concentration of Zn^2+^ not only suppresses DrHv1‐derived proton currents, but also shifts the voltage dependence and V_threshold_ to more positive membrane potentials. This implies that Zn^2+^ acts as a channel gating modifier that acts by interfering with the motion of the VSD rather than by blocking the proton conduction pathway (Cherny and DeCoursey 1999; Decoursey 2003b; Qiu et al. 2016; Okamura 2017).

We observed that DrHv1 is nearly 10 times less sensitive to extracellular Zn^2+^ than is mHv1(Fig. 4D). The IC_50_ of Zn^2+^ for DrHv1 (about 100 *μ*mol/L) is close to that for SpHv1 (Sakata et al. 2016). Similarly, several studies also reported the low Zn^2+^ sensitivity of CiHv1, cloned from *Ciona intestinalis*, which contains the same homologous Zn^2+^‐binding residues as found in DrHv1 (Sasaki et al. 2006; Qiu et al. 2016). Collectively, these results indicate that the Zn^2+^ sensitivity of Hv1 varies among species, and high Zn^2+^ sensitivity (~1 *μ*mol/L) may be restricted to a select subpopulation among vertebrates.

A previous study demonstrated that His193, in the S3‐S4 linker region is essential for Zn^2+^‐binding to hHv1, and mutation of this residue alleviated the inhibitory effect of Zn^2+^ (Ramsey et al. 2006). Because His193 of hHv1 is conserved as His189 in mHv1, but not in DrHv1, and mHv1 maintains high Zn^2+^ sensitivity, it seems likely that replacement of the His189‐homologous residue with Asp in DrHv1 (Fig. 5A) interferes with Zn^2+^‐binding and thus decreases the sensitivity of DrHv1 to extracellular Zn^2+^.

Likewise, the homologous His was replaced by Asp in the African clawed frog (*Xenopus laevis*) and selected teleosts, though not the channel catfish, where His was substituted with Asn. Considering the present results with DrHv1, we anticipate that Hv1 in these species also shows lower Zn^2+^ sensitivity than mammalian Hv1s, though further analysis is necessary. Interestingly, our data listed in Table 2 suggest there is a correlation between the total serum Zn^2+^ concentration and the presence of a Zn^2+^‐coordinating His in the S3–S4 linker region of the Hv1 channel. In humans (Yanagisawa 2004; Flomenbaum et al. 2006) and mice (this study), whose Hv1 orthologs contains a Zn^2+^‐coordinating His, serum Zn^2+^ concentrations are comparatively low. In contrast, in the African clawed frog (this study), zebrafish (this study), and channel catfish (Cort et al. 1995), whose Hv1 orthologs lack a Zn^2+^‐coordinating His, serum Zn^2+^ concentrations are comparatively high. This may also be related to the difference in the Zn^2+^ sensitivity of Hv1 orthologs in the different animal species.

Prior studies noted the importance of Zn^2+^ for the proper physiological function of Hv1 in human spermatozoa (Lishko et al. 2010) and mammalian macrophages (Subramanian Vignesh et al. 2013). The high Zn^2+^ sensitivity of mammalian Hv1 is necessary for normalization of their functions. Therefore, the acquisition of higher Zn^2+^ sensitivity by Hv1 in a subpopulation of vertebrates, including mammals, may have occurred to compensate for the lower Zn^2+^ concentration in their blood. In the case of zebrafish, whereas the Zn^2+^ sensitivity of DrHv1 is about an order of magnitude lower than that of mHv1, the serum Zn^2+^ concentration is about 8 times higher in zebrafish than in mouse. It is therefore still possible that the Zn^2+^ sensitivity of DrHv1 significantly affects its physiological function in native tissue. Although the physiological effect of Zn^2+^ has not been extensively investigated in zebrafish, our results demonstrate the importance of Zn^2+^ homeostasis as well as a link between higher Zn^2+^ concentration and lower Zn^2+^ sensitivity of Hv1 in teleost species.

## Conclusion and perspective

We have explored for the first time the characteristics of the Hv1 ortholog in zebrafish from the viewpoints of molecular structure and functional expression in tissue. In particular, we focused on the electrical activity in zebrafish neutrophils and their sensitivity to extracellular Zn^2+^. Our study revealed the conserved expression of *hvcn1* in neutrophils among diverse vertebrates and also highlighted the variation in Zn^2+^ sensitivity of Hv1 orthologs in the different species, which may correlate with their serum Zn^2+^ concentrations. We anticipate that further analysis will provide additional insight into the physiological significance of Zn^2+^‐sensitivity as well as its importance to the function of Hv1 channel in diverse species.

## Conflict of Interest

None declared.

## Data Accessibility

## Supporting information




**Figure S1:** Multiple amino acid alignment of full‐length Hv1 from human (*Homo sapiens*, hHv1), mouse (*Mus Musculus*, mHv1), zebrafish (*Danio rerio*, DrHv1), sea squirt (*Ciona intestinalis*, CiHv1), sea urchin (*Strongylocentrotus purpuratus*, SpHv1), insect (*Nicoletia phylophila*, NpHv1), and two species of dinoflagellates (*Lingulodinium polyedrum*, LpHv1; *Karlodinium veneficum*, kHv1). Putative transmembrane helices (S1‐S4), the short amphipathic helix S0, and the coiled‐coil domain are indicated in the boxes. Zn^2+^‐coordinating residues are shown in red. The model of DrHv1 topology (lower panel) was generated using TOPO2 Transmembrane Protein Display public online software (http://www.sacs.ucsf.edu/cgi-bin/open-topo2.py/)
**Figure S2:** Multiple amino acid alignment of full‐length Hv1 from human (*Homo sapiens*, hHv1), mouse (*Mus Musculus*, mHv1), African clawed frog (*Xenopus laevis*, XlHv1), Medaka (*Oryzias latipes*, OlHv1), Pufferfish (*Tetraodon nigroviridis*, TnHv1), Tilapia (*Oreochromis niloticus*, OnHv1), zebrafish (*Danio rerio*, DrHv1), and channel catfish (*Ictalurus punctatus*, IpHv1). Putative transmembrane helices (S1‐S4), the short amphipathic helix S0, and the coiled‐coil domain are indicated in the boxes.
**Figure S3:** (A) Representative proton current traces recorded from mHv1‐ and DrHv1‐expressing HEK293T cells (left and right, respectively; the same traces as shown in Figure 1B) were fitted by a single exponential function (red broken line). The red arrowheads indicate the points where the normalized current–voltage relationships were calculated. (B) Normalized current–voltage relationships of mHv1 calculated from the activation phase of actual proton currents (empty circles; *n* = 5) and fitted results (filled circles; *n* = 5). (C) Normalized current–voltage relationships of DrHv1 calculated from the activation phase of actual proton currents (empty squares; *n* = 5) and fitted results (filled squares; *n* = 5). Data are presented as means ± SD. Note that in (B) and (C) the data calculated from actual proton currents and fitted results are highly equivalent, both in mHv1 and DrHv1.
