# Supplementary material for: Molecular and functional characterization of the voltage‐gated proton channel in zebrafish neutrophils
Source: Physiol Rep. 2017 Aug 3;5(15):e13345. doi: 10.14814/phy2.13345 (PMC5555884; doi:10.14814/phy2.13345)
Supplement: Supplementary file 1 — Figure S1: Multiple amino acid alignment of full‐length Hv1 from human (Homo sapiens, hHv1), mouse (Mus Musculus, mHv1), zebrafish (Danio rerio, DrHv1), sea squirt (Ciona intestinalis, CiHv1), sea urchin (Strongylocentrotus purpuratus, SpHv1), insect (Nicoletia phylophila, NpHv1), and two species of dinoflagellates (Lingulodinium polyedrum, LpHv1; Karlodinium veneficum, kHv1). Putative transmembrane helices (S1‐S4), the short amphipathic helix S0, and the coiled‐coil domain are indicated in the boxes. Zn2+‐coordinating residues are shown in red. The model of DrHv1 topology (lower panel) was generated using TOPO2 Transmembrane Protein Display public online software (http://www.sacs.ucsf.edu/cgi-bin/open-topo2.py/) Figure S2: Multiple amino acid alignment of full‐length Hv1 from human (Homo sapiens, hHv1), mouse (Mus Musculus, mHv1), African clawed frog (Xenopus laevis, XlHv1), Medaka (Oryzias latipes, OlHv1), Pufferfish (Tetraodon nigroviridis, TnHv1), Tilapia (Oreochromis niloticus, OnHv1), zebrafish (Danio rerio, DrHv1), and channel catfish (Ictalurus punctatus, IpHv1). Putative transmembrane helices (S1‐S4), the short amphipathic helix S0, and the coiled‐coil domain are indicated in the boxes. Figure S3: (A) Representative proton current traces recorded from mHv1‐ and DrHv1‐expressing HEK293T cells (left and right, respectively; the same traces as shown in Figure 1B) were fitted by a single exponential function (red broken line). The red arrowheads indicate the points where the normalized current–voltage relationships were calculated. (B) Normalized current–voltage relationships of mHv1 calculated from the activation phase of actual proton currents (empty circles; n = 5) and fitted results (filled circles; n = 5). (C) Normalized current–voltage relationships of DrHv1 calculated from the activation phase of actual proton currents (empty squares; n = 5) and fitted results (filled squares; n = 5). Data are presented as means ± SD. Note that in (B) and (C) the data ca [file PHY2-5-e13345-s001.pdf]

Figure S1

```
hHv1   MAT-----------------------WDEKAVTRR-AKVA----------------------------------------  16
mHv1   MTS-----------------------HDPKAVTRR-TKVA----------------------------------------  16
DrHv1  M-------------------------------------------------------------------------------  1
CiHv1  MEGDNCNKSRHKSHNMI------NPNYASVRCTQPLPSVIQLRSRNKMIGITEDPSSDSEPVSSNQ------PLLLTNLS  68
SpHv1  MKPD-LKQTEEVSQGLLEQGDGGSSSHGNNKVGEK-SGDGQFAEQGGVCETSEDKNDGCETHSGAKTDKHKTKLEGETAP  78
NpHv1  MWL-----------------------------------------------------------------------------  3
LpHv1  MAG-----------------------------------------------------------------------------  3
kHv1   M-------------------------------------------------------------------------------  1

                                                                                  S0
hHv1   --PAERMSKFLRHFTVVGDDYHAWNINYKKWENEEEEEEEEQPPPTPVSGEEGRAA-APDVAPAPGPAPRAPLDFRGMLR  93
mHv1   --PTKRMSRFLKHFTVVGDDYHTWNVNYKKWENEEEEEE---PAPTSAEG-EGNAE-GPDAEAGSASTPRQSLDFRSRLR  89
DrHv1  -------SRYLKHFTAVGDNKNA----VPTWHEEDTSHH-----------VTTLHD-APDGLEVSTGQHLGQLSFRDSLR  58
CiHv1  YEVHTFNDNNNHERPAPQEQSTQ--NTMISMQSEQKSDR--FTASNLGMFQYMKFEIGEDGDDHEEE---AILTNREKLR  141
SpHv1  ADAEMFGFRRLSDTTKPSEGNDQ----QRVIVKDDSSDS-------------VVSD-SHDGHPARTE----PLSLREKLH  136
NpHv1  ---KMDAHKRLSE------------DLEKVIMKEDGNSS-------------IMTEPDHNIQPSK--------TVRERLR  47
LpHv1  -----------HH---------------------------------------GAPS-KHEEHAATAPH-----GIKHGL-  26
kHv1   -------DRILHH---------------------------------------AVHT-VHTSKSARDAE--GHGTWQSKLN  32

                           S1
hHv1   KLFSSHRFQVIIICLVVLDALLVLAELILDLKIIQ---------------------------------------------  128
mHv1   KLFSSHRFQVIIICLVVLDALLVLAELLLDLKIIE---------------------------------------------  124
DrHv1  KLYSTERFQIVVVCLVVLDAIFVLCELLIDLSIIE---------------------------------------------  93
CiHv1  HILHSKPIHVAIIVLVVLDSFLVVGELLIDLKVII---------------------------------------------  176
SpHv1  EIMETQKFHIAILVLVVIDCILVIVELVIDFEVLSQEEGQCNATETD---------------------------------  183
NpHv1  KLLHSHKFQISVITLVIIDCLLVITELLIDLEM-----------------------------------------------  80
LpHv1  QLYNSKACLVVLFFLLILDVCIVVASGVLETHYLISKADDCKAYVDACPHGHGRRLDAPRPWSGLGAAGAEAAPSPGRRL  106
kHv1   EALNSSKVHTILNVLLICDLMTVIIGMLLEQYYSDSQVQGLTEAFKDCLE---KRTFCPDP-------------------  90

                                                                       S2
hHv1   -------------------------------------------PDKNNYAAMVFHYMSITILVFFMMEIIFKLFVFRLEF  165
mHv1   -------------------------------------------PDEQDYAVTAFHYMSFAILVFFMLEIFFKIFVFRLEF  161
DrHv1  -------------------------------------------ADHHRIAPQVFHYLSLALLTFFMVELAGKIFAYRLEF  130
CiHv1  -------------------------------------------VPHGNPAPEILHGFSLSILSIFMVEIALKIIADHRHF  213
SpHv1  ------------------------------------------KEEKEVTAANVLHYISIGILSIFMIELLIKIPVFRMEF  221
NpHv1  -------------------------------------------HEEESLAQHVLHYCSITILSIFIVEIFLKLYAFRQEF  117
LpHv1  DGLDAADAEASSGGGLFLAGHDAGRQLSSSDGDQIDCHHPHFGNHSLHDAEKILAYISIGILVIFIIEQLLLIAAMRGAY  186
kHv1   ------------------------------------SHLAHYGNHDLHEWAERMEYASLAILLIFLLENMLLVLANGCRF  134

                 S3            ▼            S4                     Coiled-coil domain
hHv1   FHHKFEILDAVVVVVSFILDIV--LLFQEHQFEALGLLILLRLWRVARIINGI--IISVKTRSERQLLRLKQMNVQLAAK  241
mHv1   FHHKFEILDAFVVVVSFVLDLV--LLFKSHHFEALGLLILLRLWRVARIINGI--IISVKTRSERQILRLKQINIQLATK  237
DrHv1  LHHKFEVFDGIVVVVSFILDII--YISKEDAFDAMGLLILLRLWRVARIINGI--LVSVQNRANHRVEKLKEINESLVHQ  206
CiHv1  IHHKVEVLDAVVVVISFGVDIALIFVGESEALAAIGLLVILRLWRVFRIINGI--IVTVKTKADDRVHEIKKKNSELELQ  291
SpHv1  FRSKLEVFDGIIIVISFVLDVV--SLIYEEQFAVLQLLVLLRLWRIVRVVNGV--ILSVETQAKKKIEQQKHLRAEVEHE  297
NpHv1  FKHRLEVFDAIIVIVSFALDIA--FRNSRDALSGVGLIIILRLWRVARVLNGV--VLSVKMQAEHQLEREKQRGMALEGE  193
LpHv1  FREKLMVLDGFVITLSLLLEILV------TNLPLGGLLVVARIWRFARAGHGT-------MEGSHDVHKVHPVLGTFPKE  253
kHv1   FANPFHILDIVVVVVSVGFELQ--GILGEGHDAGIGLVVFARTWRFIRLGHGIHEMHEEHEAEDHGEHRVSDAAGSLEAP  212

hHv1   IQH-LEFSCSEKEQEIERLNKLLRQHGLL---------------------------------------------------  269
mHv1   IQH-LEFSCSEKEQEIERLNKLLKQNGLL---------------------------------------------------  265
DrHv1  VNE-LKEQNTKMDQENVRLRALLKDHSIDF--------------------------------------------------  235
CiHv1  IHN-LEEKLSQKEQDMSRLHEILRCNNIDIPPTVPLTTSVQIHSTTTASADV----------------------------  342
SpHv1  MEK-FRRYCAAQEKEIEVLRNTLNQHGIQIDDDYVAKKPQFSLNQLNVVVEMNSADKH-DTGEDEGEGEEGGGDGNTRRH  375
NpHv1  LSR-CRQVCAAQQRELDVLRAVLQHHGLD--------QQLPDGNRVDVVADVEKR-------------------------  239
LpHv1  LSEQVWAHMSGEKWEAMLLRNGTEKLEMDVAKEEQRIAAQIAQAHPSVVLRALAAERQRQAAREQLQAQESKASG-----  328
kHv1   LQK-----------------GSFEQHAKG--------TSGVHHARSQASSNREGRE------------------G-----  244

hHv1   -------------------------------GEVN  273
mHv1   -------------------------------GDVN  269
DrHv1  -----------------------------------  235
CiHv1  -----------------------------------  342
SpHv1  EKEREALGEHTITLTTDDNVNTIQADYHPQDTTFT  410
NpHv1  -----------------------------------  239
LpHv1  -------------------------------RPAV  332
kHv1   -------------------------------CCVQ  248
```

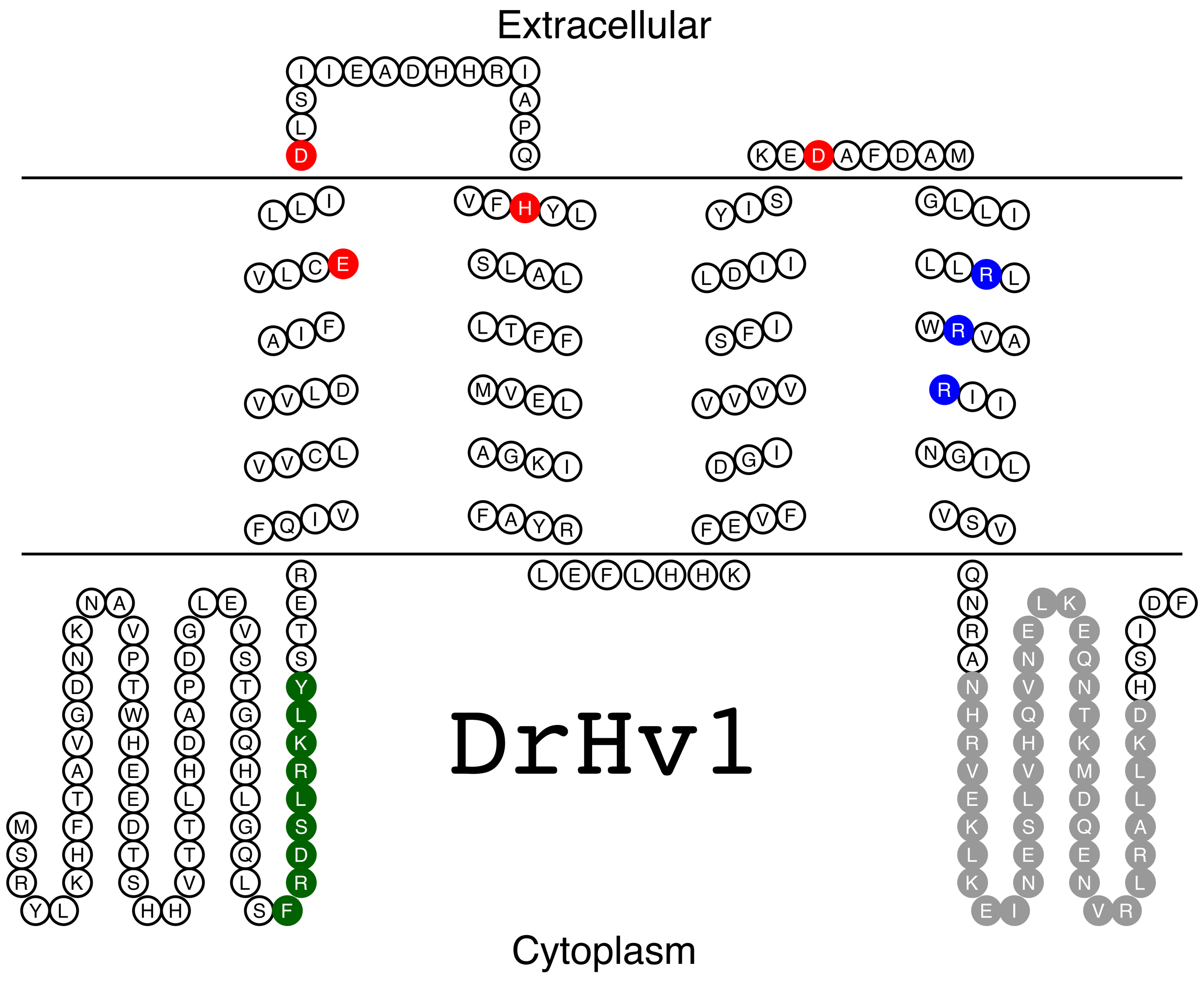

Histidine-binding residue

Figure S2

```
hHv1    MATWDEKAVTRRAKVAPAERMSKFLRHFTVVGDDYHAWNINYKKWENEEEEEEEEEQ-PPPTPVSGEEGRAAAPDVAPAPG   79
mHv1    MTSHDPKAVTRRTKVAPTKRMSRFLKHFTVVGDDYHTWNVNYKKWENEEEEEE----PAPTSAEGE-GNAEGPDAEAGSA   75
XlHv1   --------------------MAGCLRHFTSVGDDTK-----KREWKQED------------------VEVAYEEPLKNTP   37
OlHv1   --------------------MSRYLKHFSTVGDKH------HTVWEDEE------------------LHAVSED-LSPAS   35
TnHv1   --------------------MSRYLRFVTTVGDDQ------PAQWDEEE------------------LHVNSEE-LSSAT   35
OnHv1   --------------------MSRYLKHFTAVGDQH------VVHSDEEE------------------LHVSSEE-LSSAT   35
DrHv1   --------------------MSRYLKHFTAVGDNKNA----VPTWHEEDTSHH-----------VTTLHDAPDG-LEVST   44
IpHv1   --------------------MVRFLRFFTVVGDDHQA----ASRWHEED------------------LHKVSED-LEAAK   37

                 S0                  S1                              S2
hHv1    PAPRAPLDFRGMLRKLFSSHRFQVIIICLVVLDALLVLAELILDLKIIQPDKNNYAAMVFHYMSITILVFFMMEIIFKLF   159
mHv1    STPRQSLDFRSRLRKLFSSHRFQVIIICLVVLDALLVLAELLLDLKIIEPDEQDYAVTAFHYMSFAILVFFMLEIFFKIF   155
XlHv1   HPFIASYSFRGALKWLLSSHKFQIVIICLVILDALFVLVEVLLDLELLAEKVDHIIPEIFHYLSISVLTFFILEIAGKLY   117
OlHv1   GLFPTSLTFREALKRLYVSERFQVAVVCLVILDACFVLAELLIDLSVIKLDHGHIAPEVFHYLSLAVLTFFMMELAGKLF   115
TnHv1   GQYPTTLTVRERLKQMYCSERFQILVVCLVILDAIFVLVELLLDLSIIKLDHGSVAPEVFHFLSLGLVVFFLLELAGKLF   115
OnHv1   GQLPATLTFRDSFKRLYSSERFQVLVVCLVILDAIFVLAELLIDLAVIKLEHGHIAPEVFHYLSLAILTFFMLELAGKLF   115
DrHv1   GQHLGQLSFRDSLRKLYSTERFQIVVVCLVVLDAIFVLCELLIDLSIIEADHHRIAPQVFHYLSLALLTFFMVELAGKIF   124
IpHv1   PPAT-QLTFRDGLKNLFSSQRFQIAVVCLVIMDALFVLCELLLDLSIIEVDEHHIAPQVFHYLSLALLTFFIVELAFKLY   116

                    S3                 ▼          S4                  Coiled-coil domain
hHv1    VFRLEFFHHKFEILDAVVVVVSFILDIVLLFQEHQFEALGLLILLRLWRVARIINGIIISVKTRSERQLLRLKQMNVQLA   239
mHv1    VFRLEFFHHKFEILDAFVVVVSFVLDLVLLFKSHHFEALGLLILLRLWRVARIINGIIISVKTRSERQILRLKQINIQLA   235
XlHv1   AFRLEFFHHKFEVFDAAIVVISFIIDIVYISREDIFNAVGLLILLRLWRVARIVNGVIVSVKTRAEEKMHKLKEQKGSLL   197
OlHv1   AFRLEFFHHKFEMFDGLVVVVSFILDIVFIFHEDAFDGMGLLILLRLWRVARIINGIVVSVKNRADQRIHKLKESYDHVV   195
TnHv1   AFRKEFFDHKFEVFDGLVVTVSFVLDVAFIFHEDAFDGIGLLILLRLWRVARIINGILVSVKTREQQKLHKLKESYDHLV   195
OnHv1   AYHLEFFLHKFEVFDGFVVIVSFILDIVFIFHEDAFDGIGLLILLRLWRVARIINGILVSVKTRADQKIHKLKESYDHLV   195
DrHv1   AYRLEFLHHKFEVFDGIVVVVSFILDIIYISKEDAFDAMGLLILLRLWRVARIINGILVSVQNRANHRVEKLKEINESLV   204
IpHv1   AFRLEFFHHRFEVFDAIVVIVSFILDIVYISKENAFDGMGLLILLRLWRVARIINGILLSVKSQANHRVQKLKEENDRLH   196

           Coiled-coil domain
hHv1    AKIQHLEFSCSEKEQEIERLNKLLRQHGLLGEVN   273
mHv1    TKIQHLEFSCSEKEQEIERLNKLLKQNGLLGDVN   269
XlHv1   EKVAQLEQQCAQQEQEIGRLHKLLQEHNVFPAS-   230
OlHv1   QRVTELQKSCDDLTQENHRLKAILKENQIDF---   226
TnHv1   QRVTELQQLTDKLEKEKQRLQALLKKHDIEF---   226
OnHv1   QRVTELQERNDKLAQENDNLKALLKKHGIDI---   226
DrHv1   HQVNELKEQNTKMDQENVRLRALLKDHSIDF---   235
IpHv1   HQIGELKEHKTAMEQEVSKLRAVLAKHSIQY---   227
```

Figure S3

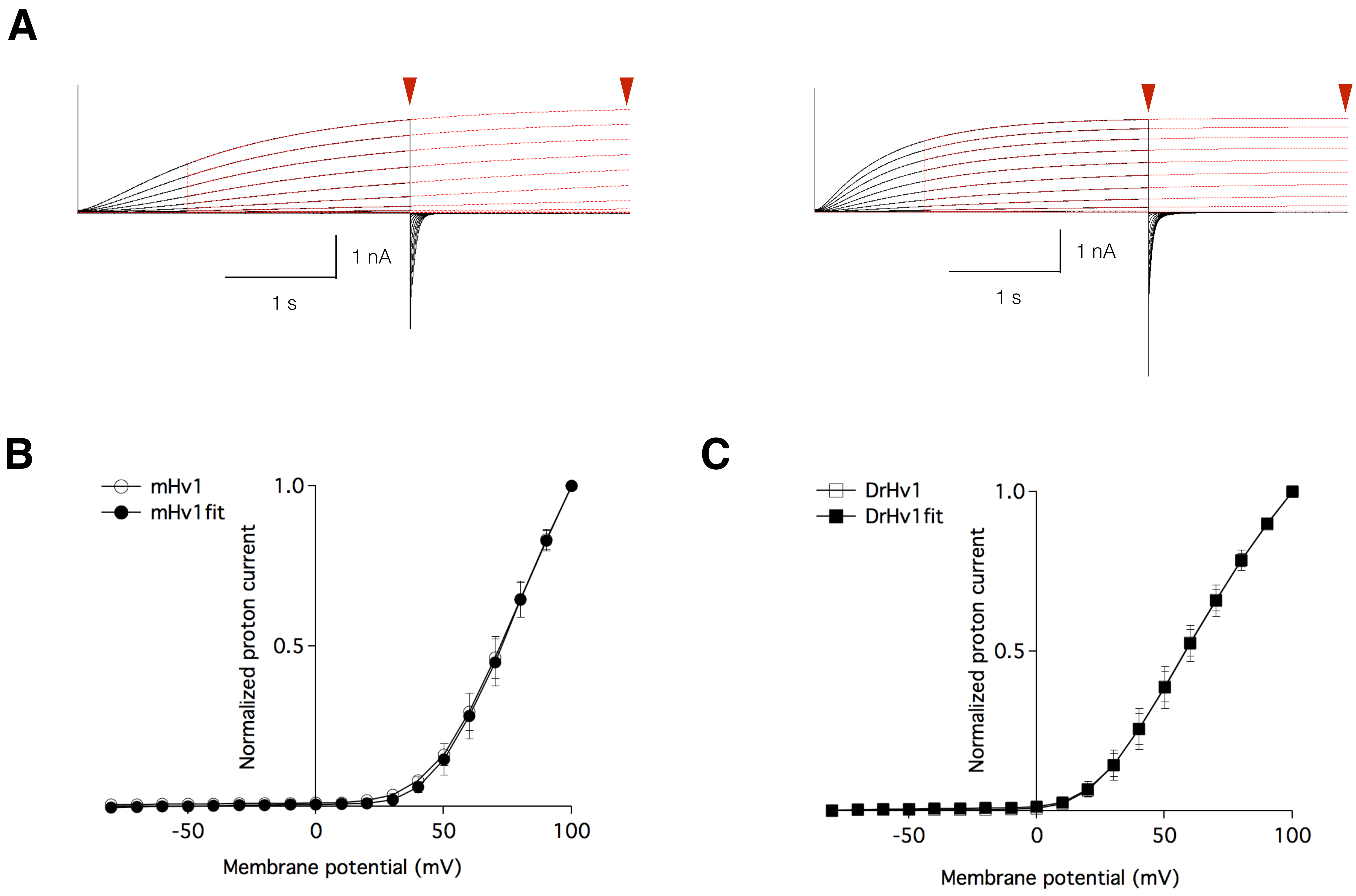
A
1 nA
1 s
1 nA
1 s
B
mHv1
mHv1fit
Normalized proton current
1.0
0.5
-50
0
50
100
Membrane potential (mV)
C
DrHv1
DrHv1fit
Normalized proton current
1.0
0.5
-50
0
50
100
Membrane potential (mV)
